# Le déficit immunitaire humoral: mieux le connaître pour mieux le prendre en charge

**DOI:** 10.11604/pamj.2014.18.272.4061

**Published:** 2014-08-04

**Authors:** Jalila El Bakkouri, Zahra Aadam, Fatima Ailal, Hanane Salih Alj, Ahmed Aziz Bousfiha

**Affiliations:** 1 Laboratoire d'Immunologie, Centre Hospitalier Universitaire Ibn Rochd, Casablanca, Maroc; 2 Faculté de Médecine et de Pharmacie, Université Hassan II, Casablanca, Maroc; 3 Laboratoire de Recherche Biologie et Santé, Faculté des Sciences Ben M'sik, Université Hassan II, Casablanca, Maroc; 4 Unité d'Immunologie Clinique, Centre Hospitalier Universitaire Ibn Rochd, Casablanca, Maroc

**Keywords:** Déficit immunitaire humoral, agammaglobulinémie, déficit immunitaire commun variable, déficit en IgA, Humoral immune deficiency, Agammaglobulinemia, common variable immunodeficiency, IgA deficiency

## Abstract

Les déficits immunitaires humoraux (DIH) sont des maladies hétérogènes allant des formes asymptomatiques rencontrés lors des déficits sélectifs en immunoglobulines A (IgA) et en sous-classes d'IgG aux formes graves des agammaglobulinémies congénitales. Les patients atteints de DIH présentent souvent des infections ORL ou des voies respiratoires récidivantes ou sévères. Ces patients peuvent présenter un certain nombre de complications non infectieuses, telles que des manifestations auto-immunes et des entéropathies, qui pourraient être le seul symptôme clinique révélateur. Les formes sévères des DIH sont facilement diagnostiquées grâce au dosage des IgG totaux, des IgA et des IgM. La thérapie substitutive par les immunoglobulines reste le traitement de choix chez ces patients.

## Introduction

Les déficits immunitaires primitifs (DIP) comprennent au moins 200 maladies héréditaires qui sont censés être individuellement et collectivement rares [1]. La prévalence et l′incidence exactes des DIP restent incertaines, mais de récentes études épidémiologiques ont suggéré que les DIP sont beaucoup plus fréquents qu′on ne le pense généralement. En Afrique, jusqu′à 902 631 personnes pourraient avoir un DIP, alors que seuls 1 016 cas sont actuellement inscrits au registre africain [2]. Actuellement, au Maroc, plus de 400 cas sont répertoriés dans le registre marocain des DIP mais il semblerait que le nombre de patients atteints de cette maladie au Maroc est au moins 5 fois cette valeur [3]. Le sous-diagnostic de cette pathologie serait dû à une certaine méconnaissance des DIP, à leur expression clinique très hétérogène et leur exploration souvent réputée difficile. Parmi ces déficits immunitaires primitifs, Les déficits immunitaires humoraux (DIH) sont de loin les plus fréquents [4]. En contraste avec la majorité des déficits immunitaires primitifs impliquant d'autres composantes du système immunitaire, les DIH peuvent se manifester à tout âge avec des pics dans l'enfance et dans la troisième décade de la vie [5, 6]. La conscience de ces notions est fondamentale pour établir un diagnostic rapide et initier le traitement approprié. Il est important de distinguer les déficits primitifs en anticorps de plusieurs états de déficits immunitaires secondaires dont les tumeurs lymphoïdes malignes et l'infection par le VIH [7].

Ces DIH représentent un groupe hétérogène de pathologies d’étiologies diverses. Leur conséquence commune finale est l'incapacité à produire une réponse immunitaire efficace contre les agents pathogènes invasifs. L'hétérogénéité de la pathogénie de ces affections peut produire des tableaux cliniques nettement différents, bien qu'il existe de nombreuses similitudes entre ces différents syndromes [7]. Un défaut de production des anticorps a comme conséquence des infections récurrentes et/ou sévères. Les DIH se présentent par un spectre hétérogène de symptomatologie clinique, allant le plus souvent des formes asymptomatique dans les déficits sélectifs en IgA et en sous-classes d′IgG aux agammaglobulinémies congénitales sévères dans lesquelles la production de tous les isotypes d′immunoglobulines est sévèrement diminuée [4].

La majorité des patients atteints de DIH symptomatiques présentent des infections ORL et/ou des infections respiratoires récurrentes et sont difficiles à dépister parmi les nombreux enfants qui se présentent en pratique pédiatrique. Mis à part les infections récurrentes, il ya un large éventail d′autres complications cliniques associées à ces déficits immunitaires primaires [6, 8, 9], affectant la qualité et l'espérance de vie de l′enfant.

Cet article vise à fournir aux biologistes et aux cliniciens un aperçu sur ces pathologies qui restent sous- diagnostiquées. Après une introduction sur le développement des cellules B normales, nous décrirons la physiopathologie, le diagnostic et le traitement des différents DIH.

## Rappel: Développement et maturation des lymphocytes B

**Figure 1:**
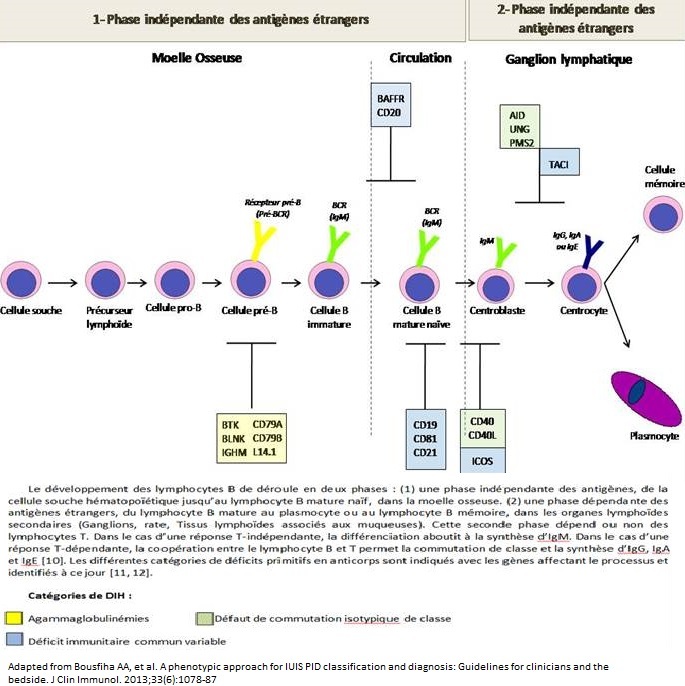
Etapes de développement les lymphocytes B

Un répertoire correct d'immunoglobulines est nécessaire pour une protection efficace de l'hôte contre les différents agents pathogènes. Les immunoglobulines (Ig) sont secrétées par les plasmocytes qui représentent le stade final de différenciation des lymphocytes B. Ces derniers se développent à partir des cellules souches hématopoïétiques (précurseurs lymphoïdes) de la moelle osseuse où ils subissent une série de différenciations durant lesquelles survient le réarrangement des gènes du récepteur des lymphocytes B (BCR - B cellreceptor). Ces BCRs sont représentés par un complexe de signalisation associé à une molécule d'IgM membranaire. Ces BCRs sont capables de reconnaître une grande variété d′antigènes. L'expression réussie de la première chaine lourde Mu et ultérieurement de la chaine légère (Kappa ou Lambda) sur la surface de la cellule B immature permet la poursuite de la différenciation du stade pré-B au stade de cellule B mature naïve. Cette cellule quitte la moelle osseuse et rejoint le pool des lymphocytes B circulantes. Ces événements sont résumés sur la Figure 1 [10, 11].

Après avoir quitté la moelle osseuse, la rencontre du lymphocyte B avec l'antigène, dans les ganglions lymphatiques régionaux, initie une série supplémentaire de différentiations de lymphocytes B aboutissant à la production des immunoglobulines. En effet, suite à l'interaction entre le lymphocyte B et le lymphocyte T spécifique à l'antigène, la commutation isotypique mène à la substitution des IgM initiaux par des IgG, IgA ou des IgE de haute affinité. Le rôle initial fondamental des IgM réside dans la liaison avec les agents pathogènes intravasculaires et dans l'activation du complément, tandis que la réponse immunitaire secondaire, caractérisée par la production des immunoglobulines type IgG, IgA et IgE, est nécessaire pour une protection continue et efficace contre les agents pathogènes invasifs. Les IgA accomplissent de multiples fonctions dans la défense des muqueuses. Dans le milieu extravasculaire, les IgG constituent la clé effectrice du processus d'opsonisation et d'activation du complément. Les IgE sont impliquées dans la défense contre les parasites. La production des IgG de haute affinité constitue un pilier dans la réussite de la vaccination ainsi qu'une composante capitale de la mémoire immunitaire contre différents agents infectieux. Les troubles résultant d'un déficit dans chacune des étapes citées antérieurement ont des conséquences cliniques plus ou moins graves [7, 10, 11].

Les antigènes polysaccharidiques, présents dans la paroi des bactéries encapsulées comme pneumocoques et méningocoques, induisent une réponse anticorps T-indépendante qui se produit dans la zone marginale de la rate. La réponse cellulaire B de type T-indépendante est immature chez des enfants sains de moins de 2 ans, d′où leur vulnérabilité à de graves complications aux infections à pneumocoque et à méningocoque [5].

## Pathogénie des déficits primitifs en anticorps

Un défaut qui se produit lors des étapes critiques du développement des cellules B a le potentiel de causer des DIH (Figure 1), mais les défauts immunologiques et génétiques de la plupart des patients atteints de la DIH sont encore inconnues [12]. Chez un enfant présumé atteint d'un DIH, il est faut éliminer les causes secondaires d'une carence en anticorps, tels que le syndrome néphrotique, l'entéropathie exsudative, l′utilisation de certains médicaments, et les tumeurs malignes hématologiques. Selon la nature du défaut des cellules B, les DIH peuvent être séparées en différentes catégories (Tableau 1), avec leurs propres caractéristiques cliniques, immunologiques et génétiques. Dans cette section, nous discuterons des différents DIH plus en détail, y compris les spécificités de diagnostic et les plus importantes complications cliniques.

**Tableau 1:** Les types de déficit immunitaire humoral et leurs caractéristiques

Type de DIH	Ig du sérum	Lc B circulants	Pathogénie	Transmission	Signes associés
Agammaglobulinémies congénitales :	Très diminué (tous les isotypes)	Diminué	Blocage du développement des lymphocytes B au niveau de la moelle osseuse	Liée à l'X ou AR	Infections respiratoires et ORL récidivantes. Parfois neutropénie concomitante.
Défaut de commutation isotypique de classe d'immunoglobuline	IgM : Normaux ou augmenté IgG et IgA : Diminué	Lymphocytes B à IgG, A et E absents	Anomalie co-stimulation des cellules B et T au niveau du centre germinatif	lié à l'X	Infections respiratoires et ORL récidivantes Infections opportunistes Neutropénie
Déficit immunitaire commun variable (DICV)	IgG : Diminué IgA et/ou IgM : Diminué	Normaux ou Diminué	Défaut d'activation des LcB, de costimulation ou de survie des LcB	?	Infections récurrentes respiratoires et ORL Cytopénies autoimmunes Signes digestifs
hypogammaglobulinémie transitoire de l'enfant	IgG : Diminué IgA et IgM : Normaux ou Diminué	Normaux	?	?	Asymptomatique Infections récurrentes respiratoires et ORL Signes digestifs Varicelle sévère – candidose buccale
déficit sélectif en anticorps : IgA, IgG2 et anticorps anti-polysaccharides	- IgA: Diminué - IgG2 : Diminué - Absence de réponse antineumococcique	Normaux	?	?	Asymptomatiques si isolés Infections respiratoires et ORL récurrentes Infections respiratoires et ORL récurrentes Diarrhées – Giardiase Allergies – maladies autoimmunes Infections bactériennes

### Les agammaglobulinémies congénitales

Le premier cas rapporté d′agammaglobulinémie congénitale date de 1952 [13], lorsque Bruton décrit le cas d'un garçon présentant des infections récurrentes et un déficit en gammaglobulines. Plusieurs années plus tard, il est apparu que les garçons avec agammaglobulinémie liée à l'X (XLA- X-linkedagammaglobulinemia) souffrent d′une anomalie du gène codant pour la tyrosine kinase de Bruton ou BTK (Bruton's tyrosine kinase) [14]. Cette enzyme est nécessaire pour la signalisation du (pré) BCR. Une carence en BTK entraîne un blocage précoce du développement des cellules B dans la moelle osseuse, entraînant l′absence (ou presque) des lymphocytes B du sang périphérique et les tissus lymphoïdes périphériques. En conséquence, la production de tous les isotypes des immunoglobulines, y compris la réponse à la vaccination est fortement diminuée. XLA représente 85% des cas d'agammaglobulinémie congénitale [5].

Les patients atteints de formes autosomiques récessives ont aussi des anomalies des cellules B qui affectent le pré-BCR ou en aval de la cascade de signalisation. Les manifestations cliniques de ces patients sont similaires à l'XLA, mais le phénotype clinique a tendance à être plus sévère en raison d′un blocage plus complet du développement précoce des cellules B [15].

Plus de la moitié des patients XLA ont des manifestations cliniques avant la 1ère année d′âge mais plus de 90% ne sont diagnostiqués qu’à l′âge de 5 ans [16]. Moins de 10% des patients présentent des symptômes dans les 3 premiers mois en raison de la protection par les anticorps maternels transférés par voie placentaire. Les infections ORL et les infections respiratoires récidivantes sont les symptômes les plus fréquents, mais les enfants peuvent également présenter des infections bactériennes sévères d′autres localisations de l'organisme [16]. Mis à part un déficit immunitaire sévère, 11% des enfants atteints de XLA souffrent de neutropénie concomitante, ce qui peut être diagnostiqué à tort comme une neutropénie congénitale. Conformément à la stratégie de diagnostic pour les enfants présentant des infections ORL et des infections respiratoires récurrentes, l'agammaglobulinémie congénitale peut être facilement évoquée devant les taux faibles d′immunoglobuline IgG,IgA et IgM [17]. L'analyse des sous-populations lymphocytaires révèlera par la suite que les cellules B sont sévèrement diminuées dans le sang périphérique. Au cas où les cellules B sont présentes, autres DIHs doivent être évoqués, en particulier les hypogammaglobulinémies transitoires de l′enfance et les anomalies de commutation de classe (voir ci-dessous). Quand les cellules B sont absentes, un diagnostic définitif peut être fait par l′analyse génétique des gènes candidats. Le traitement consiste en une thérapie substitutive par les immunoglobulines et un traitement antibiotique empirique des infections bactériennes. Si une neutropénie est présente, elle disparaît avec la thérapie substitutive par les immunoglobulines. A long terme, la maladie pulmonaire obstructive chronique est la plus fréquente complication. Les patients XLA sont sensibles à la méningo-encéphalite chronique à entérovirus, qui est une cause importante de décès [16].

### Défaut de commutation isotypique de classe d'immunoglobuline

Ces anomalies, anciennement appelés syndromes hyper IgM, sont des affections rares caractérisées par une diminution du taux sérique des IgG et IgA, associée à un taux normal ou élevé d′IgM [18]. Le mécanisme en cause est soit une anomalie de co-stimulation des cellules B et cellules T au niveau du centre germinatif, affectant l′initiation de la commutation isotypique, ou un dérèglement du processus de commutation isotypique de classe elle-même. Le prototype d′un défaut de co-stimulation est le déficit en CD40-ligand lié à l′X [19–22]. Le déficit en CD40L provoque non seulement un DIH, mais aussi une anomalie fonctionnelle des cellules T puisque ce déficit en anticorps est secondaire à la réduction de l′expression du CD40L sur les lymphocytes T. Par conséquent, le déficit en CD40L est aujourd′hui essentiellement considéré comme une anomalie des lymphocytes T [18]. En raison de l'anomalie touchant les cellules T, ce DIH a une différence importante avec les autres DIH qui est la survenue d′infections opportunistes. En plus des pneumonies bactériennes qui surviennent chez 80% des enfants, 41% présentent des pneumonies à Pneumocystisjirovecii [22]. Le déficit immunitaire combiné sévère (SCID) fait partie des diagnostics différentiels du déficit en CD40L [23], mais contrairement à la plupart des cas de SCID, l′analyse des sous-populations lymphocytaires chez les patients atteints de DIH montrera un taux normal des lymphocytes T. Comme les patients atteints de XLA, la neutropénie peut être présente et les patients CD40L partagent également une susceptibilité accrue à la méningo-encéphalite à entérovirus. En outre, les patients CD40L sont sujets à une cholangite sclérosante fatale secondaire à une infection par Cryptosporidiumparvum. Le traitement initial consiste à administrer des immunoglobulines au patient et une prophylaxie à Pneumocystisjiroveci, mais en raison de la fréquence élevée des complications potentiellement mortelles avant l′âge de 25 ans [24], la greffe de cellules souches hématopoïétiques est actuellement le traitement de choix [25].

L'anomalie du processus de commutation isotypique est causée par un défaut de certains gènes: AID (activation-inducedcytidinedeaminase - la cytidine désaminase induite par activation) [26], UNG (uracile-N-glycosylase) [27], NEMO [28], et PMS2 [29]. Certains ont des caractéristiques dont:

La dysplasie anhidrotique ectodermique liée à l'X avec déficit immunitaire secondaire à des mutations dans NEMO donnerait une immunodéficience beaucoup plus large en plus du défaut de commutation isotypique de classe d'immunoglobuline [30] dans ce journal. - Le déficit immunitaire lié aux mutations des gènes de l′AID et l'UNG est causé par un dysfonctionnement limité à la lignée des lymphocytes B. Ces patients sont vus habituellement à un âge plus avancé que les patients présentant un déficit en CD40L. Mis à part des infections récurrentes, ils souffrent souvent d'hyperplasie lymphoïde, d'une maladie inflammatoire de l′intestin, et d'auto-immunité [27, 31].

### Déficit immunitaire commun variable (DICV)

Le déficit immunitaire commun variable (DICV) est un déficit immunitaire idiopathique dont la prévalence est estimée à 1/25000 [5]. Il est défini par des taux d′IgG sériques en dessous de 2 DS par rapport aux valeurs normales en présence d′une diminution des IgA et/ou des IgM, des infections récurrentes, une altération de la réponse à la vaccination, avec une élimination des causes définies précédemment d'hypogammaglobulinémie et un âge supérieur à 2 ans [32]. Un groupe considérable de patients souffrent d′une hypogammaglobulinémie idiopathique similaire, mais ne remplissent pas tous les critères de diagnostic. Ces patients sont généralement diagnostiqués comme DICV «possible» ou comme ayant un désordre “DICV-like”. Leurs caractéristiques cliniques se confondent avec ceux DICV. Moins de 10% des patients atteints de DICV ont des cas similaires dans la famille [6] et un défaut génétique n'a été identifié que chez moins de 10% des patients qui ont été signalés à la base de données européenne des déficits immunitaire primaires (ESID-European Society of Immunodeficiencies) comme ayant un phénotype clinique de DICV [4]. Les anomalies en cause signalées impliquent un défaut d'activation des lymphocytes B (déficit en CD19 [33] et en CD81 [34]), de co-stimulation (anomalie du récepteur ICOS - Inducible Co-Stimulatory Receptor [35]) et de survie des cellules B (déficit en BAFF-R [36]). En outre, d'autres anomalies génétiques ont été identifiées mais celles-ci ne causent pas une vraie hypogammaglobulinémie mais une simple augmentation de la susceptibilité aux maladies (déficit TACI [37–41]). L′hétérogénéité des manifestations immunologiques et cliniques du DICV gêne la découverte des mécanismes de la maladie causale, des facteurs pronostic et des anomalies génétiques sous-jacente. La plupart des patients DICV sont diagnostiqués à l’âge adulte jeune, mais les symptômes apparaissent dès l′enfance dans plus de la moitié des cas [9]. En conséquence, un retard diagnostic de plus de 5 ans est la règle [6, 42]. Parfois le DICV est précédé par un déficit en IgA, en sous-classe d'IgG, ou parun déficit en anticorps spécifique anti-polysaccharide. La symptomatologie des DICV est diverse, mais les infections récurrentes des voies respiratoires et ORL sont présentes chez plus de 90% des patients. Certains patients présentent une maladie auto-immune, le plus souvent de type cytopénies auto-immunes. Les autres manifestations cliniques sont représentées par une inflammation granulomateuse des poumons et du tractus gastro-intestinal, une diarrhée chronique inexpliquée, et des cancers hématologiques, qui sont des causes importantes de décès. Les patients atteints de DICV qui souffrent d'au moins une complication non infectieuse ont une mortalité plus élevée que les patients qui ne présentent que des complications infectieuses [43]. Le traitement consiste en une substitution en immunoglobulines et un traitement antibiotique des infections. Les immunosuppresseurs sont indiqués dans certains cas associés à des manifestations auto-immunes, mais il n'existe pas de consensus sur l'utilisation de ces agents chez les patients atteints de DICV. La surveillance de la croissance est importante car un tiers des patients développe un retard de croissance [42], qui est dû aux épisodes d'infections récurrentes, mais peut aussi se développer indépendamment des complications infectieuses et être causé par une perturbation de l′axe de l'hormone de croissance [44, 45]. Une association chez le patient d'une petite taille à un déficit immunitaire peut aussi avoir son origine d'une forme syndromique d'immunodéficience primaire [30].

### Hypogammaglobulinémie transitoire de l′enfant

L'hypogammaglobulinémie transitoire de l′enfant (HTE) doit être considérée comme un diagnostic d’élimination chez chaque jeune enfant avec hypogammaglobulinémie. Dans l'HTEon retrouve un faible taux d′IgG (<2 DS en dessous de la moyenne pour l′âge), avec ou sans diminution des IgA ou des IgM alors que les autres causes d′hypogammaglobulinémie ont été exclus [46]. La physiopathologie de ces HTE est inconnue. Chez certains patients, l'HTE pourrait être une variante de l'hypogammaglobulinémie physiologique secondaire à la disparition des IgG maternelles de la circulation qui se produit normalement à l’âge de 3 à 6 mois. De nombreux cas passent sans doute inaperçues car elles sont asymptomatiques. Parmi les cas symptomatiques, la plupart des enfants présentent des infections ORL et des infections respiratoires récurrentes avant l′âge de 1 an. D'autres présentent des diarrhées récurrentes, une infection sévère par le virus de la varicelle, ou une candidose buccale prolongée [46]. Les symptômes les plus rares sont les sepsis [47] et les méningites [48]. Chez plus des deux tiers des enfants, le taux d′immunoglobulines se normalise avant l′âge de 2 ans, mais chez certains enfants, l'hypogammaglobulinémiepeut persister jusqu′à l′âge de 5 ans [49]. Ces enfants sont traités par une antibioprophylaxie, une thérapie substitutive en immunoglobuline est généralement réaliséechez les patients présentant des infections graves ou fréquentes, malgré l'antibioprophylaxie.

### Le déficit sélectif en anticorps: IgA, IgG2 et anticorps anti-polysaccharides

Ces trois DIH sont les plus fréquents chez l'enfant et ont tendance à apparaître en association. Ces déficits lorsqu'ils sont isolés sont souvent asymptomatiques, mais leur association conduit à une immunodéficience cliniquement parlante. Cela implique que si un de ces déficits est diagnostiqué, il est utile de rechercher les autres.

Le déficit sélectif en IgA (DsIgA) est défini comme une diminution des taux sériques d′IgA < 2 DS par rapport aux témoins de même âge. La prévalence du DsIgA en Europe varie entre 1/163 et 1/875 [50, 4]. Bien que la cause des DsIgAsoit inconnue, des mutations de type TACI augmente la prédisposition à la maladie, de façon similaire au DICV [39]. Les IgA sécrétoires, sécrétée sous forme dimérique, est l′immunoglobuline de premier plan dans les sécrétions luminales des voies respiratoires et gastro-intestinales et est une composante importante de l′immunité des muqueuses. Les IgA sécrétoires ne peuvent pas être mesurés dans le sérum, mais le taux sérique desIgA monomériquesdonne un reflet indirect du niveau des IgA sécrétoires dans le corps. Le diagnostic du DsIgA est habituellement fait par dosage du taux d′immunoglobulines lors d'un bilan d'infections respiratoires récurrentes. Cependant, le DsIgA est souvent retrouvé de “façon accidentelle” dans le cadre d′un bilan de la maladie coeliaque, d'une allergie ou d'une maladie auto-immune. L′évolution clinique est asymptomatique chez de nombreux patients. Si les enfants ont des signes cliniques, généralement c'est des infections ORL et des infections respiratoires récurrentes. Les patients atteints de DsIgA sont particulièrement à risque de diarrhées chroniques et de giardiase en raison de la baisse de l′immunité muqueuse. En outre, le DsIgA est associée à une plus forte prévalence des allergies/atopies et des maladies auto-immunes, dont les cytopénies auto-immunes [50–52].

Les quatre sous-classes d′IgG se différencient par la structure de leurs régions constantes. Parmi les déficits en sous-classes d′IgG, la carence en IgG2 est celle qui est cliniquement la plus pertinente. Une diminution du taux d'IgG1 seule ne peut être considérée comme un déficit en sous-classe d'IgG puisqu'une diminution de l′IgG1 se manifeste normalement par une hypogammaglobulinémie. La plupart des patients atteints de déficit en IgG3 souffrent également d′une carence d'une autre sous-classe, alors que la sous-classe IgG4 est très fréquente, mais en règle asymptomatique. Chez les enfants sains, les IgG2 sont en quantité très faible durant les premières années de la vie, puis elles augmentent progressivement avec l′âge. Les anticorps dirigés contre les bactéries encapsulées sont principalement de la sous-classe IgG2, et une carence en IgG2 augmente donc la susceptibilité aux infections par ces bactéries. Les enfants présentant ce déficit ont souvent des infections récurrentes des voies respiratoires et des infections ORL. Chez les enfants symptomatiques ayants un déficit en sous-classe d'IgG2, une carence concomitante d′anticorpsanti-polysaccharides (specific polysaccaride antibodiesdeficiency - SPAD) doit être recherchée s′ils sont âgés de plus de 2 ans [51]. Les enfants de moins de 10 ans peuvent guérir spontanément [46].

Le SPAD doit être systématiquement recherché chez des enfants qui présentent des infections respiratoires récidivantes ou des infections ORL en continue, malgré un taux normal d'IgG, d'IgA et d'IgM. Bien que sa physiopathologie soit inconnue, une carence en CD20 a été liée au SPAD chez un patient [53]. La réponse humorale aux antigènes polysaccharidiques est normalement réduite chez les enfants sains âgés de moins de 3 ans, ce qui contribue à les rendre susceptibles aux infections dues aux bactéries encapsulées. Toutefois, certains nourrissons sont capables d'avoir des réponses normales à certains sérotypes de pneumocoque [54, 55]. Après l′âge de 2 à 3 ans, les enfants devraient être en mesure de produire une réponse suffisante aux polysaccharides pneumococciques. Une réponse insuffisante après cet âge définit la présence d′un SPAD. Une sérologie pneumococcique est donc nécessaire pour le diagnostic de SPAD. Les anticorps antipneumococciques sont mesurés avant et 2 à 4 semaines après la vaccination contre le pneumocoque. Les enfants en bonne santé devraient être en mesure de répondre à au moins sept des 14 sérotypes, au-delà d′un niveau de 0,23 à 1,66 mg/l, en fonction de l′âge et du sérotype [56, 57]. En outre, une augmentation de deux à quatre fois le niveau d′anticorps avant la vaccination anti-pneumococcique est souvent utilisée comme critère supplémentaire. Si les enfants ont reçu des vaccinations antérieures par des vaccins antipneumococciques conjugués, les sérotypes présents dans ces vaccins ne peuvent pas être utilisés pour l′interprétation de la réponse anti-polysaccharide pneumococcique [56, 57], alors l′introduction des vaccins antipneumococciques conjugués dans les programmes de vaccination nationaux limite l′utilisation de ces tests pour le diagnostic de la SPAD. Les tests de mesure de la réponse anti-pneumococcique de type IgA ou IgM pourraient être en mesure de surmonter ces limitations [4]. Le traitement du DsIgA, du déficit en IgG2 ou du SPAD est fait d′une antibioprophylaxie dans les cas symptomatiques, surtout en automne et en hiver. Si les enfants présentent une bronchectasie ou des infections respiratoires basses sévères récurrentes, une supplémentation en immunoglobulines doit être envisagée, en particulier dans le cas d′une association d'un DsIgA et/ou une carence en IgG2 avec un SPAD [5]. Les données sur l′efficacité de la supplémentation en immunoglobuline dans cette indication fait défaut, mais les études cliniques sont en cours.

## La prise en charge des déficits primitifs en anticorps [5]

### La thérapie substitutive par immunoglobulines

Avant l′introduction de la thérapie substitutive en immunoglobulines, l′espérance de vie des patients atteints d′une agammaglobulinémie ou hypogammaglobulinémie était sévèrement réduite en raison de la survenue de complications comme la maladie pulmonaire obstructive chronique secondaire aux pneumonies récurrentes et à la bronchectasie. Le traitement par immunoglobulines prévient la majorité, mais pas toutes, les complications pulmonaires [58]. L'efficacité thérapeutique est identique qu'on administre les immunoglobulines par voie sous-cutanée ou intra-veineuse [59]. Il est tout à fait possible de réaliser ce traitement à domicile ce qui améliore la qualité de vie des patients [60].

### L'antibioprophylaxie

L′utilisation de l′antibioprophylaxie pour prévenir les infections chez les patients atteints de DIH est largement pratiquée, mais elle n′est pas encore considérée comme « evidence-dased » [5]. Il y a un besoin urgent de réaliser des études complémentaires pour résoudre ce problème puisqu'en dehors de la substitution par les immunoglobulines, l'antibioprophylaxie reste une des rares options thérapeutiques chez les patients souffrant de DIH.

### Le suivi et le pronostic des DIH

Le suivi à long terme des patients atteints de DIH bénéficiant d'une substitution par les immunoglobulines devrait être axé sur la détection précoce de la maladie pulmonaire obstructive chronique. En dehors des tests fonctionnels respiratoires réguliers, il est conseillé d′effectuer au moins un scanner thoracique chez ces patients afin d′exclure une bronchectasie ou d′autres anomalies pulmonaires [61]. Une attention devrait être accordée aux symptômes évocateurs de complications non infectieuses telles que l′auto-immunité, les granulomes, une splénomégalie et une entéropathie. Une diminution des cellules B mémoire chez les patients atteints de DICV est associée à une incidence plus élevée de complications cliniques [62, 63]. Il serait donc intéressant de surveiller cette sous-population de lymphocytes chez ces patients sans oublier d'interpréter les résultats (valeurs normales) en fonction de l’âge du patient.

## Conclusion

**Figure 2:**
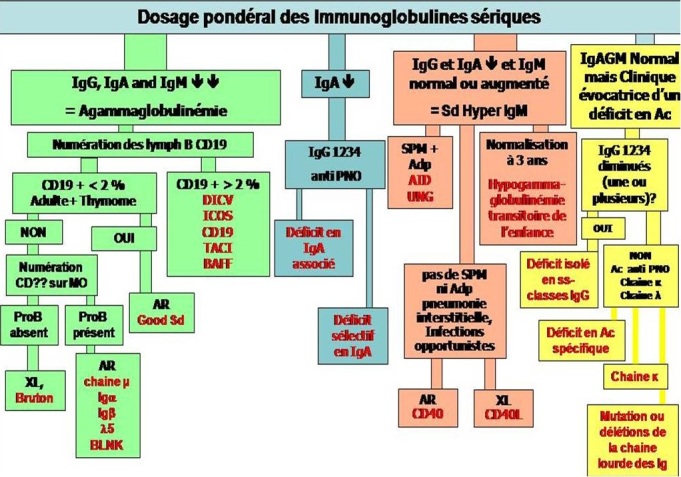
Stratégie diagnostique des déficits immunitaires humoraux [64](Adapted from Bousfiha AA, et al. A phenotypic approach for IUIS PID classification and diagnosis: Guidelines for clinicians and the bedside. J Clin Immunol. 2013;33(6):1078-87)

Les déficits immunitaires humoraux sont des maladies hétérogènes réputées d’être rares. Ils représentent le déficit immunitaire le plus fréquent chez l'adulte. Ces maladies vont des formes asymptomatiques aux formes graves des agammaglobulinémies congénitales. Ils se manifestent par des infections ORL ou des voies respiratoires récidivantes ou sévères. Ces patients peuvent présenter un certain nombre de complications non infectieuses, telles que des manifestations auto-immunes et des entéropathies, qui pourraient être le seul symptôme clinique révélateur. Ils peuvent être facilement diagnostiqués grâce au dosage des IgG totaux, des IgA et des IgM mais certaines formes nécessitent des analyses spécialisées. La thérapie substitutive par les immunoglobulines reste le traitement de choix chez ces patients. Les déficits primitifs en anticorps constituent un groupe hétérogène de pathologies dont le diagnostic précoce permet une réduction considérable de la morbidité et de la mortalité. La présence de taux normaux d'immunoglobulines sériques n'exclut pas un déficit primitif en anticorps et des investigations supplémentaires sont nécessaires dans certaines situations cliniques. Afin de rendre le diagnostic des DIH adapté aux ressources limitées de certains pays, nous rapportons la stratégie diagnostique validée récemment par les experts de l'IUIS (International union of immunological societies) [64] (Figure 2). Le diagnostic précoce des DIHréduit la morbidité et permet l'instauration d'une thérapie précoce, habituellement par substitution par immunoglobulines. Cette thérapie a prouvé ses bénéfices en cas des déficits primitifs en anticorps. Une surveillance biologique et clinique régulière de ces patients est nécessaire afin d’évaluer l'efficacité du traitement et de détecter d’éventuelles complications.
